# Protease-activated receptor 2 activation of myeloid dendritic cells regulates allergic airway inflammation

**DOI:** 10.1186/1465-9921-12-122

**Published:** 2011-09-21

**Authors:** Ian P Lewkowich, Scottie B Day, John R Ledford, Ping Zhou, Krista Dienger, Marsha Wills-Karp, Kristen Page

**Affiliations:** 1 Department of Pediatrics, Cincinnati Children's Hospital Medical Center, Cincinnati, Ohio, USA; 2 Department of Pediatrics, University of Cincinnati, Cincinnati, Ohio, USA

## Abstract

**Background:**

A common characteristic of allergens is that they contain proteases that can activate protease-activated receptor (PAR-2); however the mechanism by which PAR-2 regulates allergic airway inflammation is unclear.

**Methods:**

Mice (wild type and PAR-2-deficient) were sensitized using German cockroach (GC) feces (frass), the isolated protease from GC frass, or through adoptive transfer of GC frass-treated bone marrow-derived dendritic cells (BMDC) and measurements of airway inflammation (cellular infiltration, cytokine expression, and mucin production), serum IgE levels and airway hyperresponsiveness (AHR) were assessed. BMDC were cultured, treated with GC frass and assessed for cytokine production. PAR-2 expression on pulmonary mDCs was determined by flow cytometry.

**Results:**

Exposure to GC frass induced AHR and airway inflammation in wild type mice; however PAR-2-deficient mice had significantly attenuated responses. To directly investigate the role of the protease, we isolated the protease from GC frass and administered the endotoxin-free protease into the airways of mice in the presence of OVA. GC frass proteases were sufficient to promote the development of AHR, serum IgE, and Th2 cytokine production. PAR-2 expression on mDC was upregulated following GC frass exposure, but the presence of a functional PAR-2 did not alter antigen uptake. To determine if PAR-2 activation led to differential cytokine production, we cultured BMDC in the presence of GM-CSF and treated these cells *ex vivo *with GC frass. PAR-2-deficient BMDC released significantly less IL-6, IL-23 and TNFα compared to BMDC from wild type mice, suggesting PAR-2 activation was important in Th2/Th17 skewing cytokine production. To determine the role for PAR-2 on mDCs on the initiation of allergic airway inflammation, BMDCs from wild type and PAR-2-deficient mice were treated in the presence or absence of GC frass and then adoptively transferred into the airway of wild type mice. Importantly, GC frass-stimulated wild type BMDCs were sufficient to induce AHR and allergic airway inflammation, while GC frass-stimulated PAR-2-deficient BMDC had attenuated responses.

**Conclusions:**

Together these data suggest an important role for allergen activation of PAR-2 on mDCs in mediating Th2/Th17 cytokine production and allergic airway responses.

## Introduction

It is generally accepted that allergic asthma results from an inappropriate Th2-dominated immune response to an otherwise innocuous protein. Allergens are derived from a number of sources, including plants (grasses and trees), arthropods (mite and cockroach), animals (cats, dogs), and fungi. While allergens are from diverse sources, a common characteristic of allergens is that they contain intrinsic protease activity, or are presented in the airways along with particles that contain protease activity. For example, German cockroach (GC) contains serine proteases [1], while HDM contains both the cysteine protease Der p1 [2,3] and serine proteases Der p3 and Der p9 [4]. In addition, fungal extracts of *Alternaria alternate *and *Cladosporium herbarum *[5], as well as the cat allergen *Felis domesticus *(Fel d 1) [6] contain proteolytic activity. We recently showed that the active serine proteases in GC feces (frass) played a role in regulating airway hyperresponsiveness (AHR) to acetylcholine and mucin production in a mouse model of allergic airway inflammation [7]. In addition, removal of proteases from either *A. fumigatus *[8], American cockroach Per a 10 antigen [9], Epi p1 antigen from the fungus *Epicoccum purpurascens *[10]or Cur 11 antigen from the mold *Curvularia Iunata *[11] decreased airway inflammation and airway hyperresponsiveness in mouse models. To date, the mechanism(s) by which proteases mediate their effects is unclear.

Allergen-associated proteases are thought to regulate biological effects through the activation of protease-activated receptors (PARs). PARs (-1, -2, -3, -4) are a family of proteolytically activated G-coupled receptors which, when activated, initiate a signal transduction pathway leading to transcriptional regulation. Of particular interest is PAR-2, which has been implicated in allergic diseases. To date, only a few studies have investigated the importance of PAR-2 in modulating allergic airway disease. In studies in which systemically-induced (OVA bound to alum administered by intraperitoneal injection) airway responses were compared in wild type and PAR-2-deficient mice, PAR-2-deficient mice had decreased cellular infiltration compared to controls [12]. They also showed that sensitization and challenge of PAR-2 overexpressing mice with OVA resulted in increased AHR compared to wild type mice [12]. Since OVA does not contain protease activity, this study addressed the role of endogenous proteases (i.e. mast cell tryptase) released following an initial inflammatory event. We recently confirmed a role for PAR-2 in mediating allergen-derived allergic airway inflammation [13]; however the mechanism by which PAR-2 regulated these events is currently unclear.

Dendritic cells (DC) are the most potent antigen presenting cells and are thought to bridge innate and adaptive immunity. Mucosal DCs form a dense network associated with the airway epithelium and can form long extensions into the airway lumen [14]. To date, little is known regarding the role of proteases or PAR-2 in activating DCs. One report showed that serine protease activation of PAR-2 stimulated the development of DCs from bone marrow progenitor cells [15]. Thus it is possible that protease activation of PAR-2 may be important for DC maturation, thus promoting DC's to switch from a sentinel, antigen-capturing mode to a mature antigen-presenting mode. Recent evidence suggests that specific subsets of DCs are critical not only for the initiation of allergic airway responses, but also to drive immunity (myeloid, mDC) or tolerance (plasmacytoid, pDC) [16-18]. To date, it is unclear how these subsets are regulated in the airways. Interestingly, uptake of Alexa Fluor 488-labeled OVA by DCs was enhanced when PAR-2 was activated using a selective PAR-2 agonist [19]. Clearly, protease-PAR-2 may play an important, yet undefined, role in the regulation of DC maturation, function, and activation.

In this report we investigate the role of PAR-2 in mediating the development of allergen-induced allergic airway inflammation through the activation of DCs. We confirmed the importance of functional PAR-2 for allergic airway inflammation and show that the isolated protease from GC frass was sufficient to induce AHR, increased serum IgE and a Th2 skewing phenotype when in the presence of OVA. We found that GC frass upregulated PAR-2 expression on pulmonary mDCs but failed to detect PAR-2 on pDCs. While we failed to find differences in uptake of allergen in the PAR-2-deficient mice, there was a considerable difference in T cell skewing cytokine production in the PAR-2-deficient BMDC. Finally, we confirmed that GC frass activation of wild type BMDC was sufficient to induce AHR and airway inflammation; however this response was partially dependent on a functional PAR-2 on the BMDC. These data suggest the importance of protease-PAR-2 activation of the DC in the regulation of allergic airway responses.

## Materials and methods

### German cockroach frass

The fecal remnants (frass) from one cage of German cockroaches were transferred to a sterile container and stored at 4°C. Frass were resuspended in endotoxin-free double-distilled water (2 h at 4°C while rocking). Extracts were centrifuged to remove debris (10,000 g for 10 min at 4°C), supernatants harvested, and total protein was measured using the Bio-Rad Protein Assay Dye (Bio-Rad, Hercules, CA). GC frass was frozen in aliquots for use throughout the entire study. AlexaFluor-405 (Invitrogen, Carlsbad, CA) labeled GC frass (AF405-GC frass) was made according to manufacturers' specifications.

### Protease-enhancement of GC frass

GC frass was run through a size exclusion column (Sephadex G75 superfine, Amersham Pharmacia, Piscataway, NJ) and the fractions in the protease-containing peak were combined and run through a prepacked HiTrap Benzamidine FF affinity column (GE Healthcare, Piscataway, NJ). Serine proteases bind this column and are eluted using a buffer containing 20 mM para-aminobenzamidine (Spectrum Chemical Corp, Gardenia, CA). The fractions containing protease activity were combined, dialyzed against ddH_2_O and measured for protein concentration and protease activity as previously described [20]. Information regarding the amount of protein, enzymatic activity and endotoxin in the starting material GC frass and in the final column-purified protease sample is shown elsewhere [20]. The protease-enhanced GC frass was frozen in aliquots and used for the remainder of the studies.

### Animals and GC frass exposure

BALB/c and PAR-2-deficient mice were obtained from Jackson Laboratory (Bar Harbor, ME). PAR-2-C57Bl/6 mice were backcrossed for 10 generations onto the BALB/c background. For sensitization and challenge experiments, mice (6-8 weeks old) were anesthetized with ketamine (45 mg/kg)/xylazine (8 mg/kg) prior to inhalation of PBS (40 μl), LPS-free ovalbumin (OVA; 100 μg/mouse; Worthington Biochem Corp, Lakewood NJ), OVA (100 μg) plus enriched protease (0.5 units), or OVA (100 μg) plus LPS (0.1 μg/mouse; Sigma Chemical Corp, St. Louis MO #055:B5) on day 0, 14, and 21 [21]. Mice were harvested on Day 24. For the adoptive transfer of bone marrow-derived DCs (BMDC), 40 μl of BMDC (1 × 10^6 ^cells) suspension was administered via instillation into the airways of anesthetized mice. 14 d later, mice were exposed to a single intratracheal inhalation of either PBS (40 μl) or GC frass (40 μg/40 μl). In all cases, 72 h following the final inhalation, airway responses were measured. For flow cytometry studies, a single exposure to PBS or GC frass was followed by a lethal dose of sodium pentobarbital 20 h later. These studies were approved by the Cincinnati Children's Hospital Medical Center Institutional Animal Care and Use Committee.

### Airway hyperresponsiveness measurements

Allergen-induced airway hyperresponsiveness (AHR) was determined as we have previously described [22]. Briefly, mice were anesthetized 72 h after the last GC frass exposure, intubated and ventilated at a rate of 120 breaths per minute with a constant tidal volume of air (0.2 ml), and paralyzed with decamethonium bromide (25 mg/kg). After establishment of a stable airway pressure, 25 μg/kg weight of acetylcholine was injected i.v. and dynamic airway pressure (airway pressure time index [APTI] in cm-H_2_O × sec^-1^) was followed for 5 min.

### Assessment of airway inflammation

Lungs were lavaged with 1 ml of Hanks balanced salt solution without calcium or magnesium. The lavage fluid was centrifuged (1,800 rpm for 10 min), the supernatant was removed for cytokine analysis and immediately stored at -80°C. Total cell numbers were counted on a hemocytometer. Smears of BAL cells prepared with a Cytospin II (Shandon Thermo, Waltham, MA) were stained with Diff-Quick (Thermo Electron Corporation, Pittsburg, PA) solution for differential cell counting.

### Serum IgE

Animals were bled and serum isolated for total IgE levels using antibodies from BD Biosciences (San Diego, CA).

### Cytokine production

Liberase/DNase I digests of the lung were prepared to obtain single lung cell suspensions. Single cell suspensions (2.5 × 10^5^) were incubated for 72 hours in culture medium (RPMI) or in RPMI treated with GC frass (1 μg/ml) or ConA (10 μg/ml) and supernatants were analyzed by ELISA for Th2 cytokine expression as previously described [21].

### ELISAs

All ELISAs were from R&D Systems (Minneapolis, MN) and run according to manufacturer's specifications.

### Histology

Whole lungs were removed and formalin fixed. Lungs were embedded in paraffin, sectioned, and stained with haematoxylin and eosin (H&E) and Periodic Acid Schiff (PAS).

### Flow cytometry

Whole lungs were isolated from mice 20 h following exposure, minced and placed in RPMI 1640 containing Liberase CI (0.5 mg/ml; Roche Diagnostics, Indianapolis, IN) and DNase I (0.5 mg/ml; Sigma, St. Louis MO) at 37°C for 45 minutes. The tissue was forced through a 70-micron cell strainer, and red blood cells were lysed with ACK lysis buffer (Invitrogen, Carlsbad, CA). Cells were washed with RPMI containing 10% FBS, counted and plated at 500,000 cells per well in a 96 well plate. Staining reactions were performed at 4°C following incubation with Fc block (mAb 2.4G2) for 30 min. Myeloid DCs (CD11c^+^, CD11b^+^, Gr1^neg^, CD317^neg^) and plasmacytoid DCs (CD11c^+^, CD11b^neg^, Gr1^low^, CD317^+^) were quantified using anti-CD11c-APC (HL3), anti-CD11b-PE-Cy7 (M1/70), and anti GR-1-APC-Cy7 (RB6-8C5). PAR-2 expression was examined using a PE-conjugated mAb to PAR-2 (Santa Cruz, Santa Cruz, CA). Co-stimulatory molecule expression was examined using PE-conjugated mAbs to CD86 (GL1) or CD80 (16-10A1). Dead cells were excluded using 7-AAD. All antibodies (with the exception of PAR-2) were purchased from eBioscience (San Diego, CA). Data were acquired with an LSRII flow cytometer (BD Biosciences, San Jose, CA). Spectral overlap was compensated using the FACSDiVa software (BD Biosciences) and analyzed using FlowJo software (Treestar Inc, Ashland, OR).

### Isolation and development of mature, GC frass-pulsed bone marrow-derived myeloid DCs

Mice were given a lethal dose of sodium pentobarbital prior to removal of tibias and femurs. Bone marrow cells (1.5 × 10^7 ^cells per ml) were cultured on complete RPMI supplemented with GM-CSF (10 ng/ml, Peprotech, Rocky Hills, NJ). Fresh media was added along with GM-CSF (10 ng/ml) on day 3. On day 6, cells were treated with endotoxin-free PBS or GC frass (1 μg/ml) for 24 h. Cells were washed, counted and resuspended at 2.5 × 10^6 ^cells/ml.

### Quantitative real time PCR

RNA was extracted using a standard TRIzol method of phenol extraction. Total RNA is converted to cDNA by reverse transcription using the Superscript First Strand Synthesis System kit (Invitrogen, Carlsbad, CA). The PAR-2 primers are 5'-CTTAGCCTTCTTGCCAGGTG-3' and 5'-TCTCTGCACCAATCACAAGC-3' and the β-actin primers are 5'-TGTTACCAACTGGGACGACA-3'and 5'-GGGGTGTTGAAGGTCTCAAA-3'. Amplification was performed by PCR using SYBR Green on the iCycler (BioRad Laboratories) as follows: 1 cycle 95°C for 3 min, followed by 40 cycles of [95°C for 5 sec, 57°C for β-actin and 60°C for PAR-2 for 5 sec, 72°C for 10 sec], 95°C for 1 min, 55°C for 1 min and then a hold of 25°C. The target gene is normalized to the reference gene using the Pfaffl method [23].

### Statistical analysis

When applicable, statistical significance was assessed by Students t-test or one-way analysis of variance (ANOVA). Differences identified by ANOVA were pinpointed by Student-Newman-Keuls' multiple range test using SigmaStat software.

## Results

### PAR-2 promotes the development of GC frass-induced asthma

**Figure 1:**
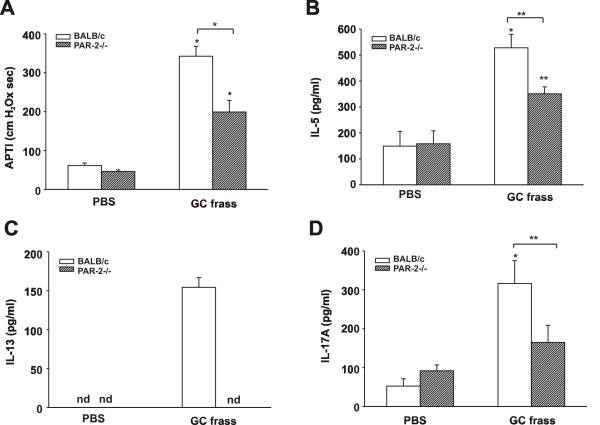
**Reduced allergen-induced AHR in PAR-2-deficient mice**. BALB/c or PAR-2 -/- mice were treated with PBS or GC frass (40 μg/40 μl) GC frass on days 0 and 14. Mice were sacrificed on Day 17. In all cases the data are expressed as mean ± SEM and represent 4-6 mice per group and statistical significance determined by ANOVA (in all cases * p < 0.001, **p < 0.05). A. AHR was measured as airway pressure time index (APTI) in cm-H_2_O × sec ^-1^. B-D. Lungs from the mice were excised; cells dissociated and maintained in a single suspension culture for 3 days in the presence of GC frass (1 μg/ml). B. IL-5 levels. C. IL-13 levels. D. IL-17A levels.

To determine the role of PAR-2 in mediating allergen-driven AHR, wild type and PAR-2-deficient mice were exposed to GC frass by intratracheal instillation on day 0 and 14, and three days later airway responses were measured. Exposure of wild type mice to GC frass resulted in increased AHR which was significantly attenuated in PAR-2-deficient mice (Figure 1A). Next we cultured cells from whole lungs and re-stimulated the cells with GC frass to assess the ability of GC frass to induce cytokine production. GC frass induced production of the Th2 cytokines IL-5 (Figure 1B), IL-13 (Figure 1C) and IL-4 (data not shown) and the Th17 cytokine IL-17A (Figure 1D). Importantly, PAR-2-deficient mice had significantly reduced production of the Th2 cytokines and IL-17A. GC frass exposure also induced a significant increase in eosinophilia and neutrophilia into the BAL fluid of wild type mice, and this was also significantly reduced in the PAR-2-deficient mice (Table 1). Together these data suggest an important role for PAR-2 in regulating allergic airway inflammation.

**Table 1:** Differential cell count in BAL fluid of wild type and PAR-2-deficient mice.

	Mac	Epi	Eos	Neut	Lymph
BALB/c PBS	1.4 ± 0.2	1.7 ± 0.4	0	0.04 ± 0.02	0.09 ± 0.05
PAR2-/- PBS	1.6 ± 0.3	3.5 ± 1.6	0	0.02 ± 0.02	0.07 ± 0.03
BALB/c frass	8.6 ± 3.6	5.2 ± 1.6	1.7 ± 0.5	4.4 ± 0.7 *	1.6 ± 0.04
PAR2-/- frass	3.4 ± 1.1	4.0 ± 1.5	0.6 ± 0.3 †	1.2 ± 0.02 †	1.5 ± 0.8

BALB/c and PAR-2-/- mice were challenged by intratracheal inhalation on Day 0 and 14 with PBS or GC frass (40 μg). On day 17, BAL fluid was harvested and differential cell counts performed. These data represent 4-6 mice per group and are expressed as mean ± SEM of cell number × 10^3^. Cell counts were statistically significant between GC frass and PBS exposed mice (*p < 0.05) and between BALB/c and PAR-2-/- mice (†p < 0.05).

### GC frass protease strongly promotes the development of Th2 immunity

**Figure 2:**
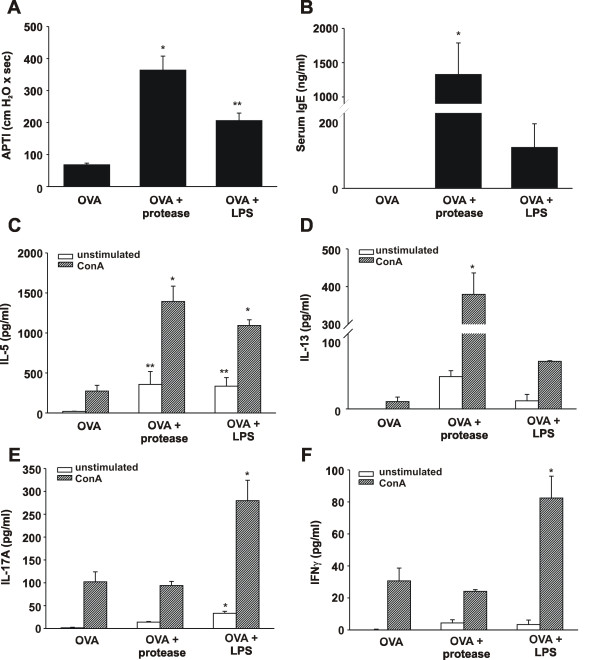
**GC frass protease is an adjuvant for OVA-induced airway responses**. Wild type mice were sensitized and challenged to LPS-free OVA, OVA plus enriched GC frass protease (OVA+protease) or OVA plus LPS (0.1 μg LPS; OVA+LPS) on Days 0, 7, and 14. On day 17, mice were anesthetized and acetycholine was injected after establishment of a stable airway pressure. In all cases the data are expressed as mean ± SEM (n = 4-6 mice per group) and statistical significant was determined by ANOVA. A. AHR was measured as airway pressure time index (APTI) in cm H_2_O × sec^-1 ^(*p < 0.001 **p = 0.015). B. Serum IgE levels were measured by ELISA (*p < 0.05). Lungs from mice were excised, cells dissociated and maintained in a single suspension culture for 3 days unstimulated or in the presence of ConA (10 μg/ml). Cell supernatants were collected, clarified and analyzed by ELISA (in all cases, *p < 0.001 and ** p < 0.05). C. IL-5 levels. D. IL-13 levels. E. IL-17A levels. F. IFNγ levels.

GC frass is a complex mixture containing both active serine proteases, and TLR4 agonists, such as LPS. Both active protease from *Aspergillus fumigatus *[8] and LPS (0.1 μg) [24] have been shown to enhance Th2 sensitization to a normally tolerogenic antigen (OVA). As such, we wished to determine the relative importance of GC frass-derived serine proteases and LPS in driving Th2 response observed following GC-frass sensitization and challenge. We have previously reported the enrichment of the active serine protease from GC frass which significantly increased the amount of protease and almost completely removed endotoxin (~1 pg/μg protein) [20]. Thus, to test whether GC frass-derived protease acted as an adjuvant for specific Th2 sensitization in the induction of allergic airway responses, we sensitized and challenged mice with OVA, OVA in the presence of GC-frass derived protease, or OVA in the presence of LPS (0.1 μg) on Days 0, 14 and 21. Importantly, while OVA itself failed to induce significant AHR, Th2, or Th17 cytokine production, OVA containing protease was sufficient to induce airway responsiveness to cholinergic agents (Figure 2A), increase serum IgE levels (Figure 2B), and induce the whole lung production of theTh2 cytokines IL-5 (Figure 2C), IL-13 (Figure 2D) and IL-4 (data not shown) alone or in the presence of ConA. IL-17A (Figure 2E) and IFNγ (Figure 2F) levels were unaltered by the protease. Thus, the principal effect of the protease is to enhance the development of Th2 immunity. In contrast, while the presence of LPS moderately enhanced AHR (Figure 2A), serum IgE levels (Figure 2B) and Th2 cytokine production (Figure 2C, D), it markedly enhanced production of IL-17A (Figure 2E) and IFNγ (Figure 2F), suggesting that LPS is capable of enhancing Th2, Th7 and Th1 responses. Together, these data support the observation that the active serine proteases in GC frass are sufficient to induce robust Th2 responses following mucosal allergen exposure in mice.

### PAR-2 is expressed on pulmonary mDCs and is regulated by allergen exposure

**Figure 3:**
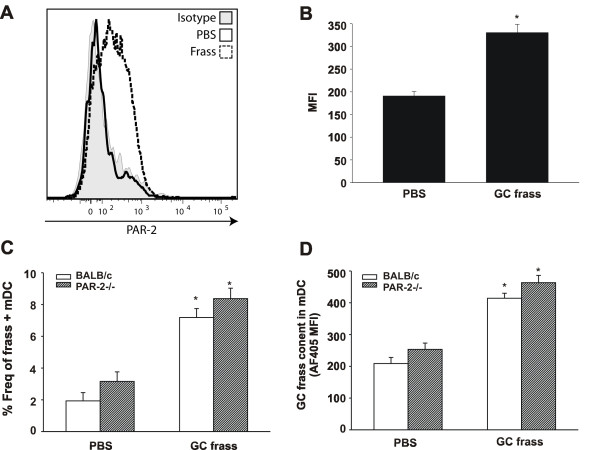
**Frass treatment increased expression of PAR-2 on pulmonary myeloid dendritic cell**. Lungs from PBS or GC frass-treated mice were harvested 20 h post exposure and stained for flow cytometric analysis of PAR-2 expression. A. Representative histogram showing PAR-2 expression on gated pulmonary mDCs from PBS-treated (solid line) or GC frass-treated (dashed line) mice. Solid grey histogram depicts staining with isotype control mAb. B. Average PAR-2 MFI on gated pulmonary mDCs. Data are expressed as Mean ± SEM (n = 6 mice per group) and statistical significance determined by ANOVA (*p < 0.001). C +D. AF405-labeled GC frass was instilled in the airways of wild type or PAR-2-/- mice and 20 h post exposure lungs were isolated and stained for flow cytometry. C. Percent of AF405-positive mDC in the lung (*p < 0.05). D. Mean fluorescence intensity (MFI) of AF405 in mDCs (*p < 0.05).

As uptake of allergen by pulmonary DCs is required for optimal stimulation of T cell responses leading to asthma, and both PAR-2 and active serine proteases are effective at enhancing Th2 responses *in vivo*, we wished to determine if PAR-2 could be detected on the surface of pulmonary mDCs. To this end, wild type mice were exposed to a single intratracheal inhalation of PBS or GC frass. Twenty hours later, whole lungs were harvested, dissociated and stained for flow cytometric analysis of PAR-2 expression on pulmonary DCs. PAR-2 expression was apparent on neutrophils (data not shown); however PAR-2 expression on pulmonary mDCs and pDCs could not be reliably detected in naïve mice. Importantly, inhalation of GC frass induced the expression of PAR-2 on the pulmonary mDCs (Figure 3A, B) but not on pDCs (data not shown). This suggests the upregulation of PAR-2 on mDCs but not pDCs following allergen exposure.

One possibility for the decreased allergic airway inflammation in PAR-2 could be differential uptake of allergen in DCs between wild type and PAR-2-deficient mice. To address this, wild type or PAR-2-deficient mice were exposed to a single intratracheal inhalation of PBS or Alexa405-labeled (AF405) GC frass. Twenty-four hours later, whole lungs were harvested, dissociated and stained for flow cytometry. We found no difference in the percentage (Figure 3C) of AF405-GC frass positive mDCs or in the MFI (Figure 3D) between wild type and PAR-2-deficient mice exposed to the allergen. Taken together these data demonstrate that while PAR-2 expression is enhanced on pulmonary mDCs after GC frass exposure, it does not mediate its Th2-enhancing properties by enhancing antigen uptake by pulmonary mDC.

### Differential cytokine production from PAR-2-deficient BMDCs

**Figure 4:**
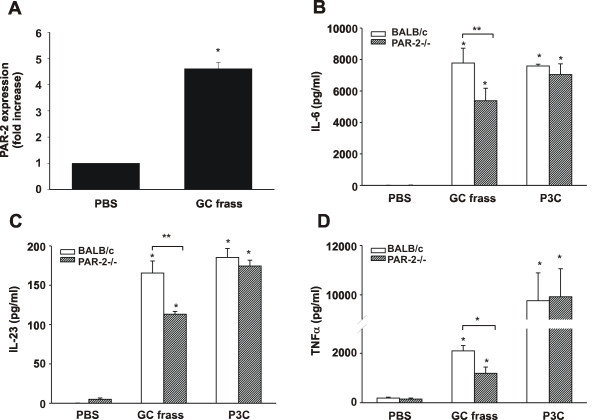
**GC frass-induced PAR-2 mRNA and cytokine production from BMDC**. A. Bone marrow was isolated from BALB/c mice and cultured in the presence of GM-CSF for 6 days. Cells (5 × 10^6^) were cultured in the presence or absence of GC frass (1 μg/ml) for 4 h. Cells were extracted in TRIzol, RNA was synthesized and converted to cDNA. Quantitative real time PCR was performed. PAR-2 was normalized to β-actin and levels are expressed as fold-increase over control (mean ± SEM for 4 separate experiments; *p < 0.001). B-D. Bone marrow was isolated from BALB/c and PAR-2-deficient mice and was cultured in the presence of GM-CSF for 6 days. Cells (1 × 10^6^) were cultured in the presence or absence of GC frass (1 μg/ml) or Pam3Cys (100 ng/ml) for 18 h. Cell supernatants were harvested, clarified and analyzed for cytokine expression by ELISA. In all cases data are expressed as means ± SEM for 4 separate experiments. B. IL-6 expression (*p < 0.001, **p < 0.05). C. IL-23 expression (*p < 0.001; **p = 0.002). D. TNFα expression (compared to PBS *p < 0.001; compared to GC frass **p = 0.023).

To better delineate the importance of PAR-2 expression on the activity of mDCs, we cultured BMDCs from WT or PAR-2-deficient mice with GC frass for 18 hours and cytokine expression was measured by ELISA. BMDCs were > 90% mDCs as characterized by flow cytometry (CD11b^+ ^CD11c^+ ^Gr1^neg ^CD317^neg^; data not shown). As observed on pulmonary mDCs from *in vivo *GC frass treated mice, we also observed that PAR-2 expression was induced by GC frass exposure in BMDCs (Figure 4A). GC frass increased cytokine expression from wild type and PAR-2-deficient mDC; however the levels of IL-6 (Figure 4B), IL-23 (Figure 4C), and TNFα (Figure 4D) in PAR-2-deficient mDCs were significantly lower than in wild type mDCs. The levels of IL-12 and IL-10 were not above the level of detection of the assay (data not shown). To confirm that PAR-2-deficient mDCs are capable of producing high levels of cytokines, we treated cells with the TLR2 agonist, Pam3Cys. There was no difference between the wild type and PAR-2-deficient mDC in their response to a TLR2 agonist. These data suggest that activation of PAR-2 plays a role in enhancing the ability of mDCs to produce T cell skewing cytokines following exposure to protease-containing allergens.

### Regulation of co-stimulatory molecule expression on mDC

**Figure 5:**
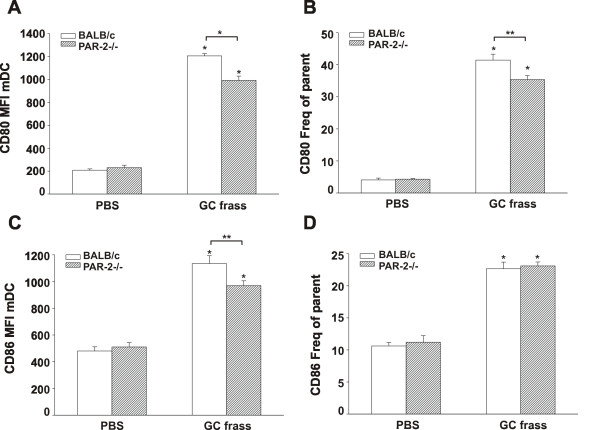
**Co-stimulatory molecule expression in wild type and PAR-2-deficient mice following exposure to GC frass**. BALB/c and PAR-2-/- mice were administered a single intratracheal inhalation of PBS (40 μl) or GC frass (40 μg/40 μl) and whole lungs were isolated 20 h later. Cells were dissociated from the lungs, and stained for flow cytometry analysis. mDC populations were analyzed for CD86 and CD80 expression. Mean fluorescent intensity (MFI) of CD80 (A) and CD86 (B) and percentage of cells expressing CD80 (B) and CD86 (D) are shown. Values are means ± SEM, n = 8 mice/group and statistical analysis was performed by ANOVA (*p < 0.001, **p < 0.05).

To further explore alterations in PAR-2-deficient mDC, we investigated the regulation of the co-stimulatory molecules CD80 and CD86 on the lung mDC population following GC frass inhalation. BALB/c and PAR-2-deficient mice were exposed to PBS or GC frass and 20 h later, whole lungs were harvested, dissociated and stained for flow cytometric analysis of CD80 and CD86 expression. GC frass increased expression of both CD80 and CD86 on mDC following a single exposure (Figure 5). There was no difference in the levels of CD80 or CD86 staining on mDCs from PBS-treated PAR-2-deficient mice compared to wild type mice, suggesting that PAR-2-deficient mice do not have inherent alterations in DC activation. Interestingly, following GC frass exposure, the number of CD86 and CD80 molecules (as indicated by MFI) on the cell surface of mDCs in PAR-2-deficient mice was less compared to wild type mice (Figure 5A and 5C). There was a significant difference in percentage of mDC expressing CD80 in the PAR-2-deficient mice compared to wild type (Figure 5B), however there was no difference in the percentage of mDC expressing CD86 between the two strains of mice (Figure 5D). This data demonstrates an overall decrease in the levels of CD80 and CD86 on mDCs in PAR-2-deficient mice and suggests that pulmonary mDCs from PAR-2-deficient mice have reduced activation and capacity for T cell stimulation following allergen sensitization.

### Adoptive transfer of PAR-2-deficient BMDC into wild type mice fails to induce allergic airway inflammation

**Figure 6:**
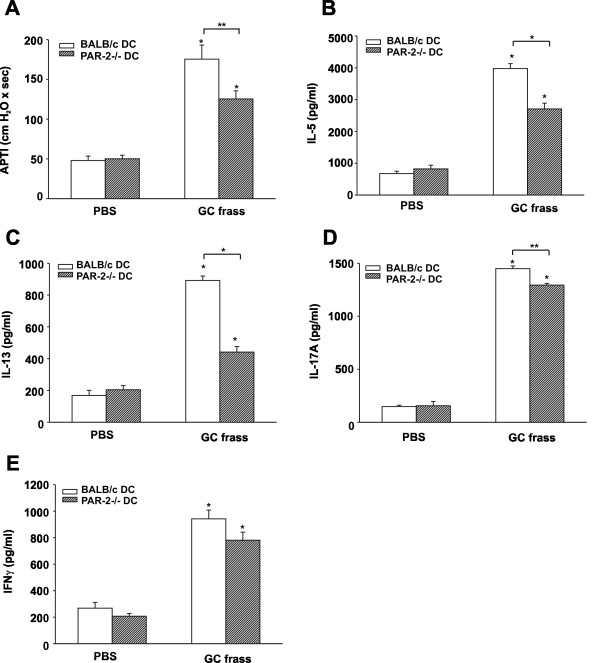
**Adoptive transfer of GC frass-pulsed PAR-2-deficient mDCs had attenuated airway responses compared to GC frass-pulsed wild type mDCs**. Naïve mice were sensitized with PBS- or GC frass-pulsed BMDC from wild type or PAR-2-/- mice on day 0. Mice were challenged with PBS (40 μl) or GC frass (40 μg/40 μl) on day 14. On day 17, mice were anesthetized and acetycholine was injected after establishment of a stable airway pressure. In all cases, means ± SEM are reported (n = 4-6 mice/group) and statistical analysis was performed by ANOVA. A. AHR was measured as airway pressure time index (APTI) in cm-H_2_O × sec ^-1 ^(* p < 0.001; **p = 0.003). Lungs from the mice were excised; cells dissociated and maintained in a single suspension culture for 3 days in the presence of GC frass (1 μg/ml). Cell supernatants were collected, clarified and analyzed by ELISA (in all cases *p < 0.001, *p < 0.05). B. IL-13 levels. C. IL-5 levels. D. IL-17A levels. E. IFNγ levels.

**Figure 7:**
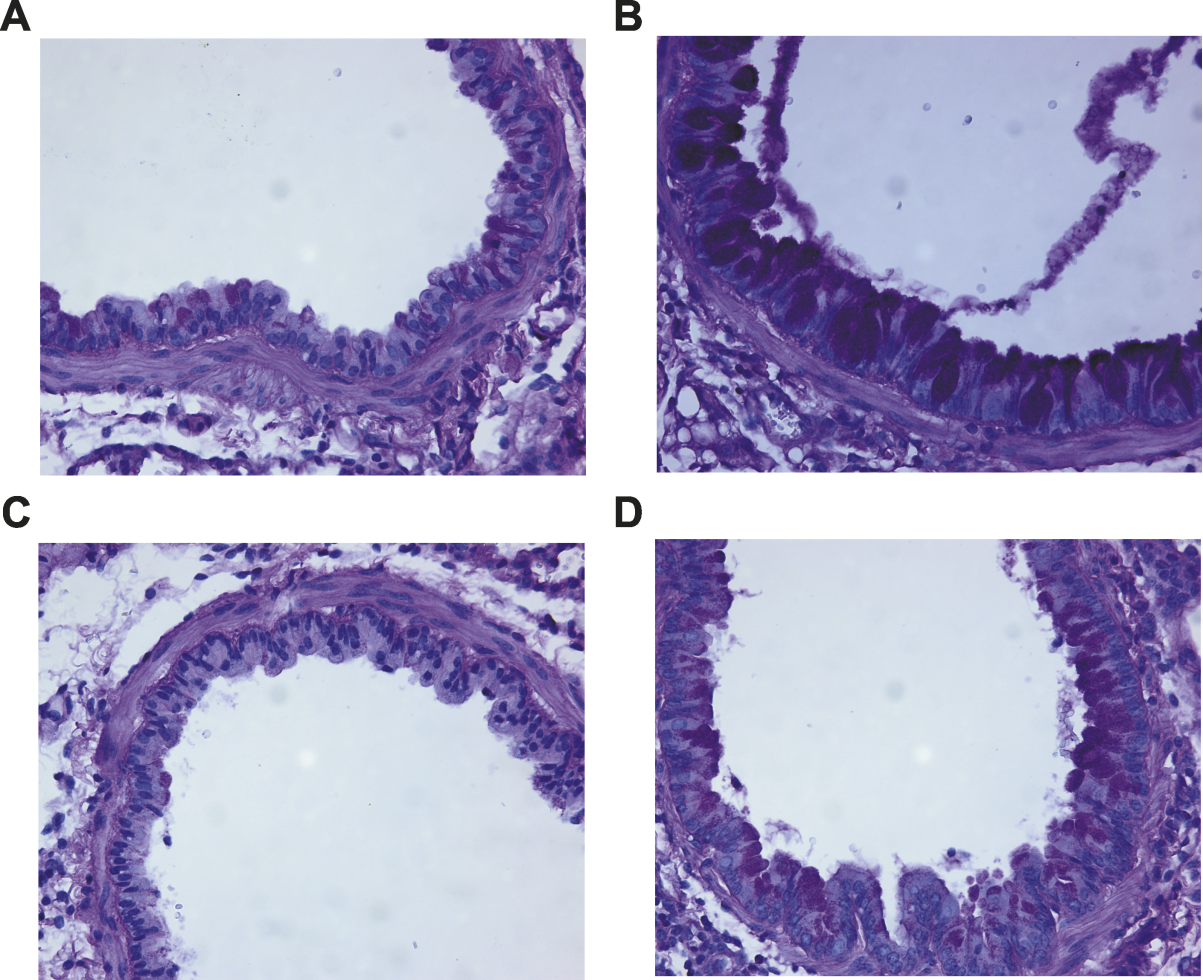
**Histological assessment of lung sections**. Naïve mice were sensitized with PBS- or GC frass-pulsed BMDC from wild type or PAR-2-/- mice on day 0. Mice were challenged with PBS (40 μl) or GC frass (40 μg/40 μl) on day 14. On day 17, lungs were isolated and fixed in formalin. Periodic Acid Schiff (PAS) staining of sectioned lungs from mice exposed to PBS-pulsed wild type BMDCs (A) or GC frass-pulsed wild type BMDCs (B). PAR-2-/- BMDC exposed to PBS (C) or GC frass (D) are also shown. Representative slides are shown of sections from 4-6 mice per group.

To further explore the role of PAR-2 on mDCs in promoting the development of allergic airway inflammation, we performed an adoptive transfer. To this end, BALB/c BMDCs were treated with PBS or GC frass *in vitro *for 18 hours and then 1 × 10^6 ^cells were adoptively transferred into the lungs of naïve BALB/c mice. Fourteen days later, mice were challenged with intratracheal inhalation of PBS or GC frass and airway responses were measured 72 h post challenge. Adoptive transfer of GC frass-pulsed wild type BMDCs induced AHR to acetylcholine challenge (Figure 6A) and increased IL-5 (Figure 6B), IL-13 (Figure 6C), IL-17A (Figure 6D), and IFNγ (Figure 6E) compared to PBS-pulsed wild type BMDCs. Thus, sensitization with GC-frass pulsed DCs induced a mixed Th1/Th2/Th17 profile similar to that observed in mice sensitized with HDM-pulsed DCs [18], and reminiscent of the profile observed in humans with more severe disease [25-28]. Importantly, adoptive transfer of GC frass-pulsed PAR-2-deficient BMDCs resulted in significantly reduced levels of AHR (Figure 6A). In addition, the restimulation of the lungs with GC frass *in vitro *resulted in up to 50% reduction in levels of Th2 cytokines (Figure 6B, C). While Th17 cytokine production was also reduced in mice sensitized with PAR-2-deficient BMDCs compared to wild type GC frass-pulsed BMDCs, the effect was comparatively mild (~10% reduction). There was no effect of adoptive transfer of allergen-pulsed PAR-2-deficient BMDC on IFNγ compared to allergen-pulsed wild type BMDC, suggesting that PAR-2 on mDCs is important in driving Th2 responses, but has limited impact on Th1 or Th17 responses. Moreover, while adoptive transfer of GC frass-pulsed mDCs from both wild type and PAR-2-deficient BMDC increased eosinophil, neutrophil, and lymphocyte infiltration in to the airways compared to PBS-pulsed DCs (Table 2), GC frass-pulsed PAR-2-deficient BMDC induced significantly less infiltration of eosinophils compared to GC-frass-pulsed wild-type BMDC. Mucin production was increased in the lungs of mice administered GC frass-pulsed wild type BMDC compared to mice that received GC frass-pulsed PAR-2-deficient BMDC (Figure 7). Administration of GC frass to either completely naïve animals or mice sensitized with PBS-pulsed BMDCs failed to initiate AHR or Th2 cytokine skewing (data not shown), suggesting the importance of the DC in the initiation of the allergic airway response, and suggesting repetition of allergen exposure is important. These data suggest that protease activation of PAR-2 on BMDC can modulate the immune response and lead to the development of allergic airway inflammation.

**Table 2:** Differential cell count in BAL fluid from wild type and PAR2-deficient mice adoptively transferred with mDCs pulsed with PBS or GC frass.

	Mac	Epi	Eos	Neut	Lymph
BALB/c DC PBS	2.2 ± 0.8	13.0 ± 3.5	0	0.1 ± 0.06	0.3 ± 0.2
PAR2-/- DC PBS	3.5 ± 1.3	14.3 ± 2.8	0	0.1 ± 0.03	0.2 ± 0.05
BALB/c DC frass	5.2 ± 2.1	9.8 ± 2.1	1.6 ± 0.4	9.5 ± 2.1 *	3.4 ± 0.5
PAR2-/- DC frass	3.8 ± 1.4	12.8 ± 3.5	0.7 ± 0.2 †	7.5 ± 1.6 *	3.0 ± 0.4

Bone marrow was isolated from BALB/c and PAR-2-/- mice and was cultured in the presence of GM-CSF for 6 days. Cells were pulsed with PBS or GC frass (1 μg/ml) for an additional 24 hours. Wild type mice were sensitized to wild type and PAR-2-/- BMDC (1 × 10^6 ^cells) pulsed with PBS or GC frass on Day 0. Fourteen days later, mice were challenged with an exposure to PBS or GC frass (40 μg/40 μl). On day 17, BAL fluid was harvested and differential cell counts performed. These data represent 8 mice per group and are expressed as mean ± SEM of cell number × 10^3^. Cell counts were statistically significant between GC frass-pulsed BMDC and PBS-pulsed BMDC (*p < 0.05) and between GC frass-pulsed wild type BMDC and GC frass-pulsed PAR-2-/- BMDC (†p = 0.015).

## Discussion

Herein we describe a mechanism whereby serine proteases promote the development of allergen-induced AHR through activation of PAR-2. Confirming our previous work [13], and that of others [29] we again demonstrate that mucosal sensitization to GC frass is reduced in mice lacking PAR-2, resulting in reduced AHR and airway inflammation. Previous studies have focused on the role for PAR-2 in direct activation of bronchial epithelial cells [20,30-32] triggering the development of innate immunity by causing the release of chemotactic factors specific for the growth and recruitment of pulmonary mDCs (i.e. CCL20, GM-CSF). We demonstrate here that *in vivo *PAR-2-deficient mice display reduced production of both Th2 and Th17-associated cytokines, suggesting that PAR-2 differentially influences multiple T cell responses. As mucosal exposure to GC frass induces upregulation of PAR-2 on pulmonary mDCs, but not pDCs, we sensitized naïve mice with GC-frass pulsed wild type BMDCs, or BMDCs from PAR-2-deficient mice. As previously observed [18], the adoptive transfer of allergen-pulsed mDCs yields robust production of IFNγ and IL-17A and airway neutrophilia along with the typical Th2 cytokines and eosinophilia, a response which more closely resembles the mixed Th1/Th2/Th17 response observed in severe asthmatics. However, in this context, the lack of PAR-2 on sensitizing DCs markedly impacts Th2 cytokine production, providing evidence that PAR-2 on mDCs is involved in promoting Th2 immune responses. In contrast, the Th17 response is only slightly diminished in mice sensitized with PAR-2-deficient DCs suggesting that PAR-2 expression on other cell types controls Th17 cytokine production. Thus, the present study suggests that specifically targeting PAR-2 activation of pulmonary mDCs may allow one to limit the development of Th2 responses at mucosal sites.

The observation that the induction of Th2 responses by GC frass is reduced in both PAR-2 deficient mice and mice sensitized with PAR-2 deficient DCs strongly implicates PAR-2 expression on mDCs on the ability of GC frass to induce a Th2-polarized immune response at mucosal surfaces. However, the mechanism whereby PAR-2 promotes the development of Th2 responses is unclear. While we observed that PAR-2 deficient BMDCs produce significantly reduced levels of all cytokines examined (IL-6, IL-23, TNFα) we have previously reported that GC frass-induced production of both IL-6 and IL-23 is completely abrogated in MyD88 -/- BMDC [33], suggesting that PAR-2 expression may amplify TLR-triggered cytokine production. However, as LPS-depleted serine proteases still demonstrated marked Th2-skewing capacity *in vivo*, it seems unlikely that the reduced Th2-skewing capacity of PAR-2 DCs is the direct result of reduced cytokine production. In contrast, PAR-2 activation has been shown to enhance maturation of BMDCs (as evidenced by increased MHC Class II and CD86 expression) [34], suggesting that reduced co-stimulatory molecule expression may be involved. In support of this possibility, we observe decreased expression of the co-stimulatory molecule CD80 and CD86 on pulmonary mDCs from PAR-2 -/- mice. Collectively, these data suggest that PAR-2 expression on pulmonary mDCs is required for optimal induction of Th2 immunity following exposure to GC frass.

While our data suggests that DCs lacking PAR-2 demonstrate reduced Th2 skewing capacity we cannot completely rule out a role for the epithelium in the ability of the GC frass to induce a Th2 response. Epithelial cells treated with allergens with active proteases (*Aspergillus *extract, Derp1) induce IL-25, a strong inducer of Th2 immunity [35] in an ERK/p38 dependent manner [32]. As we have shown that GC protease mediated induction of IL-8 from epithelial cells is dependent upon ERK activation [31], it is likely that IL-25 may also be produced. Moreover, *Alternaria *mediated PAR-2 cleavage has also been shown to induce TSLP production from bronchial epithelial cells [30]. TSLP in turn directly enhances the ability of DCs to induce a Th2 response [36,37], suggesting an additional mechanism whereby PAR-2 may amplify Th2 immunity. However, additional studies making use of mice lacking PAR-2 specifically in DC populations or pulmonary epithelial cells will be required to conclusively determine the relative contributions of PAR-2 on DCs and epithelial cells.

It is interesting to note that while there is a substantial (~75%) decrease in the number of neutrophils in the BAL in PAR-2 -/- mice, a dramatic impact on neutrophil recruitment was not observed in mice sensitized with GC frass-pulsed PAR-2 -/- BMDCs (~20% decrease). This is especially striking given that in both PAR-2 -/- and mice sensitized with GC frass-pulsed PAR-2 -/- BMDCs levels of IL-17A are only partially affected. As IL-17A is a strong promoter of neutrophilia, this is somewhat surprising. However, a recent report by Fei et al suggests that elevated IL-17A is not sufficient to drive neutrophilia in a model of allergic bronchopulmonary aspergilliosis [38]. Rather, a combination of both TNFα and IL-17A are required to get maximal neutrophil recruitment, whereas the production of IL-17A alone was associated with a more pronounced eosinophilia [38]. In this report we also demonstrate that PAR-2 expression on mDCs is required for maximal GC frass-induced production of TNFα, suggesting that differential TNFα production may explain differences in neutrophilia observed PAR-2 -/- versus mice sensitized with GC frass-pulsed PAR-2 -/- BMDCs. Indeed, in PAR-2 -/- mice, GC frass sensitization results in limited IL-5, but robust IL-17A production. Moreover, at challenge, PAR-2 -/- mice also lack DC-derived, GC frass-stimulated TNFα production resulting in a milieu with low IL-5 and TNFα, but high IL-17A levels that is not conducive to strong recruitment of eosinophils or neutrophils. In contrast, while sensitization of mice with GC frass-pulsed BMDCs also results in a low IL-5 and high IL-17A levels, GC frass challenge of these mice induces high levels of TNFα as endogenous DCs can respond to serine protease in the GC frass. This resulted in a low IL-5, high TNFα/IL-17A milieu which permits neutrophil recruitment, but resulted in limited recruitment of eosinophils.

The data presented here suggest that the initial response to inhaled GC frass is complex, and that synergy between different components is likely required for maximal allergenic capacity. However, these studies do highlight the importance of protease activity and activation of PAR-2 receptors on pulmonary mDCs in inducing the development of Th2 responses at mucosal surfaces. Taken together with a number of other studies showing the ability of proteases to induce the development of Th2 responses [30,32,39], these studies suggest that activity of proteases at mucosal surfaces may be a key factor regulating the development of Th2 immune responses. It is important to note that while there is good evidence to support that cockroach proteases and PAR-2 are involved in the Th2 responses in the airways, this study does not demonstrate the development of antigen-specific T cell responses by cockroach proteases and PAR-2. A greater understanding of the processes that lead to the development of Th2 responses may prove invaluable in therapeutic regulation of Th2 responses that are both undesirable (i.e. allergy, asthma), or desirable (parasitic clearance).

## Conclusions

The present study highlights the importance of functional PAR-2 on mDCs as critical for the development of Th2-biased immune response and indicates the mechanism by which allergen-derived proteases initiate allergic airway responses.

## List of abbreviations

AHR: airway hyperresponsiveness; BMDC: bone marrow-derived DC; ConA: concanavalin A; DC: dendritic cell; GC: German cockroach; IL: interleukin; LPS: lipopolysaccharide; MFI: mean fluorescent intensity; OVA: ovalbumin; PAR: Protease activated receptor.

## Competing interests

The authors declare that they have no competing interests.

## Authors' contributions

IPL performed the flow cytometry, participated in the design of the experiments and helped draft the manuscript. SBD participated in the design and implementation of the experiments. JRL performed the animal experiments, histology, isolated the protease, and ran ELISAs. PZ performed all the cell culture work and ran ELISAs. KD performed the AHR measurement and aided in the interpretation of results. MWK participated in the overall design of the study. KP conceived of the study, participated in its design and coordination and drafted the manuscript. All authors read and approved the final manuscript.
